# Mechanism‐Dependent Modulation of Ultrafast Interfacial Water Dynamics in Intrinsically Disordered Protein Complexes

**DOI:** 10.1002/anie.201813354

**Published:** 2019-02-28

**Authors:** Aritra Chowdhury, Sergey A. Kovalenko, Iker Valle Aramburu, Piau Siong Tan, Nikolaus P. Ernsting, Edward A. Lemke

**Affiliations:** ^1^ Structural and Computational Biology Unit Cell Biology and Biophysics Unit EMBL Meyerhofstrasse 1 69117 Heidelberg Germany; ^2^ Humboldt University Berlin Department of Chemistry Brook-Taylor-Str. 2 12489 Berlin Germany; ^3^ Biocenter Mainz Departments of Biology and Chemistry Johannes Gutenberg University Hanns-Dieter-Hüsch-Weg 15 55128 Mainz Germany; ^4^ Institute of Molecular Biology 55128 Mainz Germany

**Keywords:** intrinsically disordered proteins, nucleocytoplasmic transport, protein–protein interactions, solvation dynamics, time-resolved spectroscopy

## Abstract

The recognition of intrinsically disordered proteins (IDPs) is highly dependent on dynamics owing to the lack of structure. Here we studied the interplay between dynamics and molecular recognition in IDPs with a combination of time‐resolving tools on timescales ranging from femtoseconds to nanoseconds. We interrogated conformational dynamics and surface water dynamics and its attenuation upon partner binding using two IDPs, IBB and Nup153FG, both of central relevance to the nucleocytoplasmic transport machinery. These proteins bind the same nuclear transport receptor (Importinβ) with drastically different binding mechanisms, coupled folding–binding and fuzzy complex formation, respectively. Solvent fluctuations in the dynamic interface of the Nup153FG‐Importinβ fuzzy complex were largely unperturbed and slightly accelerated relative to the unbound state. In the IBB‐Importinβ complex, on the other hand, substantial relative slowdown of water dynamics was seen in a more rigid interface. These results show a correlation between interfacial water dynamics and the plasticity of IDP complexes, implicating functional relevance for such differential modulation in cellular processes, including nuclear transport.

Hydration is crucial to the expression of bio‐molecular functionality.^1^ Consequently, hydration dynamics are intimately related to the dynamics and function of several biomolecules, as seen in numerous examples where a correlation of hydration dynamics and functionality has been found; for example, interfacial hydration dynamics has been related to the speed of polymerases and specificity of enzymes.^2^ The role of hydration dynamics, albeit poorly understood, and dynamics in general is central to understanding intrinsically disordered proteins (IDPs), which unlike folded proteins populate a structural ensemble in their native state instead of a unique structure.^3^ As IDPs lack the luxury of rich structural elements present in folded proteins to guide molecular recognition, the molecular recognition of IDPs is largely governed by dynamical features. This makes the question of the role of hydration dynamics in IDPs a pertinent, albeit hugely neglected one. Mechanistically, IDP recognition comes in at least two core characters: coupled folding–binding and fuzzy complex formation.^4^ The former involves a process where an IDP assumes a folded structure upon binding the partner, and in the latter, IDPs retain their disorder even after partner binding. Considering the amplified importance of dynamics in IDP recognition, a fairly obvious and crucial, but still unanswered question is whether hydration dynamics play a role in governing IDP binding mechanisms. With an emphasis on solvation dynamics, here we directly interrogate the differential dynamics in two distinct IDP complexes formed via coupled folding–binding and fuzzy mechanisms using two disordered proteins, IBB and Nup153FG, both crucial players in nucleocytoplasmic transport.^5^ Our results reveal differential dynamics in these systems across timescales ranging from femtoseconds to nanoseconds, and elucidate possible molecular underpinnings and functional relevance of mechanism‐dependent differential IDP recognition.

Nup153FG is a 600aa long IDP domain that belongs to the class of FGNups, which are F‐ and G‐rich IDPs that constitute the permeability barrier of the nuclear pore complex (NPC) and facilitate transport across the NPC by binding nuclear transport receptors (NTRs).^5^ We and others have previously shown that FGNups, via FG motif facilitated ultrafast multivalent interactions, engage NTRs, such as Importinβ, without substantial conformational change and with diffusion‐limited kinetics forming fuzzy complexes.^6^ IBB (Importinβ‐binding domain of Importinα) is part of the N‐terminal disordered region of Importinα which also binds to Importinβ, but with a coupled folding–binding mechanism. While unbound IBB is an IDP, it assumes a folded helical structure in the bound state.^7^ Recognition of Importinβ by IBB is a crucial step in the formation of an import cargo complex that can traverse the NPC.^8^ At the very outset we see a clear difference in conformational changes in these two proteins upon partner binding using multiparameter single‐molecule fluorescence resonance energy transfer (smFRET) spectroscopy with pulsed interleaved excitation (PIE), which allows us to probe FRET specifically in molecules that bear both donor and acceptor dye labels and thus the distance between two labeling sites in a protein chain.6a,6c, 9 IBB (S24C/S55C) labeled site‐specifically with the FRET pair Alexa488 (donor dye) and Alexa594 (acceptor dye) showed *E*_FRET_≈0.8 which changed to ≈0.3 upon Importinβ binding (Figure 1 A,B and Figure S1). This suggested an increased distance between the labeling sites and reduced dynamics which is commensurate with the disorder‐to‐helix transition (Figure S2). Besides confirming the conformational change associated with IBB–Importinβ interaction, the experiment also largely exonerates dye labeling at these sites in IBB from interfering with binding; this is crucial as we will use S24C below to characterize the IBB–Importinβ interface. Nup153FG shows a very different behavior from IBB when binding to Importinβ. Nup153FG has a nonrandom sequence distribution of FG motifs, with a concentration of FxFG repeats in the N‐terminal region. Nup153FG labeled (see the Supporting Information for labeling details) in this region at positions S883 and S938 with the same FRET pair (Alexa 488/594) showed within experimental precision unchanged conformational dynamics and *E*_FRET_ of ≈0.7 for both bound (confirmed via fluorescence correlation spectroscopy, Figure S1) and unbound states in line with our previous studies.6a,6c

**Figure 1:**
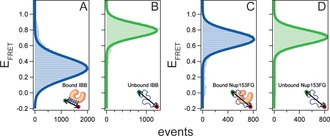
Single‐molecule histograms of FRET efficiency (*E*_FRET_). Comparison of *E*_FRET_ for IBB in Importinβ bound (A) and unbound (B) states and for Nup153FG in bound (C)and unbound (D) states. Solid lines show Gaussian fit. See Figure S1 for further experimental data.

To characterize the interface of Nup153FG and IBB in complex with Importinβ, we next employed steady‐state and picosecond‐resolved fluorescence encoded by site‐specifically labeled acrylodan, a thiol‐selective fluorophore that is highly sensitive to microenvironment polarity.^10^ For Nup153FG we engineered multiple single‐cysteine mutants spanning the length of the two different sequence stretches in the protein which are rich in FxFG motifs (883C and 990C) and PxFG motifs (1330C, 1355C and 1391C), to check for the effect of sequence propensity (Figure 2 A). For the much smaller IBB, we used the site S24C, which is at the base of the helix formed upon Importinβ binding, directly probing the interface pointing towards Importinβ (Figure 2 B). We saw large blue‐shifted acrylodan emission and increased fluorescence lifetime in both the Nup153FG mutants and IBB upon Importinβ binding (Figures S3 and S4). The acrylodan emission spectrum is highly sensitive to the local polarity with more red‐shifted emission indicating more polar environment and vice versa.^10^ Thus the blue‐shifted emission upon Importinβ binding indicated a less polar environment at the interface compared to the surfaces of free IBB or Nup153FG. This also manifested in a drop in the calculated apparent relative permittivity by ≈12 units in the bound states from the free ones. (Figure 2 C and Figure S5). As relative permittivity is a direct comparative measure of environment polarity, this likely resulted from desolvation, that is water release, from the IDP surface when the interface formed upon partner binding. Interestingly enough, the extent of inferred desolvation, and thus the associated entropic gain, upon partner binding was similar for IBB and Nup153FG, despite the fact that the binding mechanism and the structure of the IDP in the bound state are very different.

**Figure 2:**
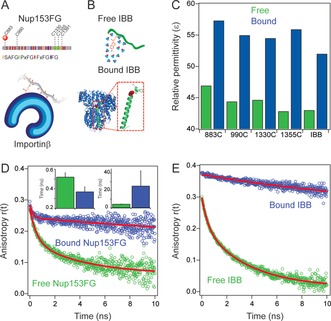
A,B) Schematic representation of Nup153FG sequence composition with labeling sites indicating interaction of acrylodan‐labeled Nup153FG with Importinβ as well as labeled IBB and the Importinβ‐bound structure (PDB: 1QGK) (labeling site highlighted with red dot). C) Relative permittivities of IBB and different sites in Nup153FG in free (green) and Importinβ (blue) bound state (see the Supporting Information for details on the permittivity calculation). D,E) Comparison of time‐resolved anisotropy in the free (green) and Importinβ‐bound (blue) states for Nup153FG 883C (D) and IBB (E). The red lines show fits to the data (bi‐exponentials for all except IBB‐bound state which was fitted with a mono‐exponential). Inset in (D) shows two time constants from global fitting of anisotropy data at five sites in Nup153FG.

We next employed picosecond‐resolved anisotropy experiments, which probe the depolarization kinetics of a fluorophore on a picosecond–nanosecond timescale. For IDPs this provides information about segmental motion, that is, the conformational motion of a segment of the chain (several amino acids), and information about very local dynamics in the immediate vicinity of the labeling site (very few amino acids), as we have shown before.^11^ Here we used acrylodan, which due to its very small size combined with a very short linker enables highly sensitive anisotropy studies. Both acrylodan‐labeled IBB and Nup153FG in the free state showed bi‐exponential relaxation behavior (Figure 2 D,E) with a nanosecond and sub‐nanosecond component and some residual anisotropy. Likely the nanosecond component related to segmental motion, while the sub‐nanosecond component related to local dynamics and the much slower hydrodynamic rotation of the entire molecule manifested itself in the residual anisotropy. For Nup153FG upon addition of Importinβ the fast sub‐nanosecond component remained and the nanosecond component became slower together with an increase in residual anisotropy (Figure 2 D, Figure S6, and Table S1). A global fit of the anisotropy decays at the tested 5 different sites of Nup153FG with two rotational correlation times showed that the longer time constant from segmental motion slowed down from ≈5 ns to ≈25 ns. Intriguingly, the faster component appeared to become slightly faster (from ≈0.52 ns to 0.37 ns) (Figure 2 D, Figure S6, and Table S1). The slowing down of the segmental motion at different Nup153FG sites and increase in residual anisotropy by ≈0.1 for 883C (Figure 2 D, Table S1) provided direct evidence of Importinβ binding. The tentative acceleration of the fast picosecond component indicated increased picosecond motion at multiple sites upon Importinβ binding. We speculate that such behavior originated from many FG motifs anchoring on the multivalent Importinβ sites and this might prevent self‐interaction of the IDP chain yielding greater flexibility around the labeling site (very short length scale). Such behavior could also compensate for the entropic penalty of binding caused by the slowed segmental motion of the chain at comparatively longer length scales. For IBB, in the presence of Importinβ, the sub‐nanosecond component completely vanished and the entire decay was described by a single component of ≈30 ns that likely originated from the hydrodynamic tumbling motion of the entire complex (Figure 2 E and Figure S7). This indicated a very rigid interface in the IBB–Importinβ complex where all picosecond motions were frozen. Due to the limited time resolution of ≈100 ps, we likely underestimated the initial anisotropy whenever ps dynamics were present (i.e. unbound state), explaining the increased initial anisotropy in the bound state that lack such dynamics (Figure 3 E). These experiments highlight a stark difference between a labile interface with picosecond motion in the Nup153FG–Importinβ complex and a more rigid and dynamically frozen interface in the IBB–Importinβ complex.

**Figure 3:**
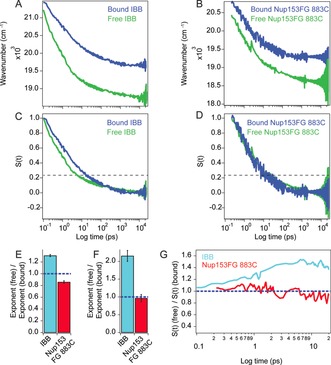
Solvation dynamics in free IDPs and complexes. A,B) TDSS from femtoseconds to nanoseconds (see the Supporting Information for details of the methods) of acrylodan in IBB (A) and Nup153FG 883C (B) in free (green line) and Importinβ‐bound (blue line) states. C,D) Normalized correlation function *S*(*t*) obtained by normalizing the data in (A) (for C) and (B) (for D) from the earliest resolved experimental time point until the Stokes shift converged ≈10 ns. The dashed line is placed at 25 % of *S*(*t*). E) Ratio of the exponents of power law fits for unbound and bound states in (A) and (B) for IBB (cyan bar) and Nup153FG 883C (red bar). F,G) Dynamics up to 20 ps. F) Bar plot is same as in (E) but the data is fitted only up to 20 ps. Error bars in the bar plots show propagated error of standard deviation of the fits. G) Ratio of the *S*(*t*) functions for unbound and bound states in (C) and (D) for IBB (cyan line) and Nup153FG 883C (red line). The data in (G) was smoothed by a moving average function with a window of 5 points.

We next set out to probe the modulation of surface water dynamics at the interface of IBB and Nup153FG complexed with Importinβ, compared to the unbound proteins. We employed dipolar relaxation to probe water dynamics from femtoseconds to picoseconds by monitoring the time‐dependent Stokes shift (TDSS) of the site‐specifically labeled acrylodan.^12^ To probe 5 orders of magnitude of solvation dynamics, we combined broadband femtosecond transient absorption (fsTA, ≤200 fs −2 ns) with time‐correlated single‐photon counting (TCSPC) to probe TDSS from ≤200 fs to 20 ns (Figures S8 and S9 and the Supporting Information). Broadband spectroscopy allowed us to obtain model‐free TDSS data bypassing the need for convolution‐based fitting of the kinetics at multiple wavelengths, which is typically used in solvation studies.2a,2b, 13 Our analysis avoided an artificial smoothing from model‐based fitting and retained the experimental noise in the data. The relaxation process in all cases was dominated by sub‐20 ps processes. In all experiments, ≥75 % of the Stokes shift had occurred under 20 ps (Figure 3 A–D). The Stokes shift in all our TDSS data in the bound state (for IBB and Nup153FG) was always at higher frequencies compared to the free state and the total Stokes shift was greater in the free state; this corresponds to the red‐shifted emission and the more solvated environment in the free state, respectively (Figure 3 A,B). This was in line with the conclusion from above that polarity decreased at the interface compared to the IDP surface on its own.

The relaxation functions shown in Figure 3 could not be fit with simple bi‐exponential or tri‐exponential functions, indicating rather a continuum of timescales in the solvent relaxation process. This led us to fit the relaxation functions with a simple power law type function (Figure S10) that described well the entire range of the Stokes shift and gave us a simple minimalistic empirical descriptor of the solvation process, without the need for extrapolative assumptions of a certain number of exponential terms a priori. The power law exponent changed from 0.34 in free IBB to 0.26 in the bound state, suggesting a slowdown of solvent relaxation in the IBB–Importinβ interface compared to the free IBB surface. For Nup153FG we saw a diametrically opposite behavior where the exponent changed from 0.32 to 0.37 indicating the overall relaxation to be faster in the bound state.

The nature of IDP solvation has been proposed to be distinctly different from that of folded proteins in their native states.^14^ While we cannot ascertain what molecular attributes give rise to such power law type relaxation in the IDPs that we have measured, unlike exponential relaxation behavior typically seen on folded protein surfaces, it is definitely an interesting question that warrants further systematic investigation. Power law type solvation dynamics have, however, been seen in several DNA structures and its origin has been a matter of debate.^15^ The power law exponent is a direct indicator of the timescale of the solvent relaxation process, with a higher exponent indicating a faster relaxation process and vice versa. It has to be noted that for power law type relaxation processes even a modest change in the exponent implies a tremendous change in the timescale of the whole relaxation process. A closer inspection of our TDSS indicates that up to 20 ps the dynamics in the bound and unbound states of Nup153FG 883C remained constant, in contrast to a large slowdown observed in IBB as also supported by the scaling exponents obtained from power law fits up to 20 ps (Figure 3 F). Since ≥75 % (dashed line Figure 3 C,D) of the relaxation process in all cases was completed within 20 ps, we can conclude that a large part of the water dynamics occurred on the sub‐20 ps timescale; this part remained unperturbed in Nup153FG while it was substantially slowed down in the IBB case. This suggested that overall acceleration of the Nup153FG solvation as seen from power law exponents occurred primarily from water relaxation at longer timescales. This was also evidenced by comparing the ratio *S*(*t*)(free)/*S*(*t*)(bound) of normalized relaxation functions *S*(*t*) for the bound and unbound scenarios for Nup153FG and IBB, where the ratio quickly approached values larger than one for IBB while for Nup153FG the values stayed close to one and started decreasing slowly at longer times (Figure 3 G). We also measured solvation dynamics at a different site in Nup153FG, namely 1391C, which showed a qualitatively similar trend to 883C manifested by an increase in the power law exponent in the bound state from 0.25 to 0.32 (Figure S11). While seeing differences, in terms of the dynamical attributes, at multiple sites in Nup153FG in different sequence contexts would have also been an interesting outcome, the qualitative similarity we see at the different sites was also reassuring and validates the robustness of the findings.

Lacking experimental evidence of discrete relaxation processes in the solvation dynamics, we cannot assign distinct molecular species of water molecules such as free water, bound water, coupled protein–water dynamics, etc. widely used in literature for less “fuzzy” systems.^16^ However, the results strongly suggest that while the interfacial water dynamics in IBB–Importinβ were slowed down substantially compared to the free state, for Nup153FG in the bound state most of the water dynamics remain unperturbed with some acceleration at longer times. Our experiments provide crucial insight into the very different nature of interfacial dynamics in IDP complexes depending on the mechanisms that drive their formation. A folded structure is associated with dynamic stability and consequently in the IBB–Importinβ complex formed by coupled folding–binding, the interface is dynamically rigid and the interfacial solvation dynamics is substantially retarded. In the fuzzy Nup153FG–Importinβ complex the accelerated picosecond motions seen in the time‐resolved anisotropy might well be driven by the accelerated solvation dynamics in the longer (>20 ps) timescales, considering the recently established paradigm where solvation dynamics beget conformational fluctuations of the protein.^16^ While interfacial water dynamics in any protein complex and hence ipso facto any IDP complex is hitherto unmeasured, it is still interesting to speculate to what extent this could relate to general differences in IDP binding mechanisms. In biology, coupled folding–binding complexes are often associated with kinetic stability,^3^ which can be attributed to a rigid interface and slowed interfacial solvation dynamics. Fuzzy complexes, on the other hand, are typically associated with more transient interactions, which can be more advantageous for several biological functions.^3^, 4b A dynamic interface associated with unperturbed or somewhat accelerated solvation dynamics might facilitate fuzziness. A simple activation barrier based argument can explain how slowed down interfacial solvation dynamics vs. largely unperturbed interfacial solvation dynamics might facilitate kinetic stability and kinetic lability, respectively (Figure S12). This is a question relevant to cargo transport through the nuclear pore complex, which is known to be fast, yet specific.^6^ In the case of Nup153FG and IBB, such behavior can be directly linked to their functions as well. Kinetic stability in the IBB complex is crucial to maintain the integrity of the import cargo complex during transport across the NPC, while kinetic lability is important for FGNups to facilitate fast transport. Minimal perturbation of most (≥75 %) of the solvation dynamics in FGNups poses minimal energy barriers from solvation towards unbinding events. This might be the crucial barrier‐reducing mechanism that allows FGNups to remain mobile on NTR surfaces.^6^, ^17^ We hypothesize that such drastically different interfacial solvation dynamics might be a general mechanism for IDPs to tune the plasticity of complexes ranging from kinetically stable to fuzzy, and thus encode various functionality. In summary our work underscores the supreme importance of ultrafast dynamics, especially that of the solvent milieu.

## Conflict of interest

The authors declare no conflict of interest.

## Supporting information

As a service to our authors and readers, this journal provides supporting information supplied by the authors. Such materials are peer reviewed and may be re‐organized for online delivery, but are not copy‐edited or typeset. Technical support issues arising from supporting information (other than missing files) should be addressed to the authors.

Supplementary
