# Supplementary material for: Mechanism‐Dependent Modulation of Ultrafast Interfacial Water Dynamics in Intrinsically Disordered Protein Complexes
Source: Angew Chem Int Ed Engl. 2019 Feb 28;58(14):4720–4. doi: 10.1002/anie.201813354 (PMC6563697; doi:10.1002/anie.201813354)
Supplement: Supplementary file 1 — Supplementary [file ANIE-58-4720-s001.pdf]

Angewandte Chemie
Eine Zeitschrift der Gesellschaft Deutscher Chemiker

Supporting Information

Mechanism-Dependent Modulation of Ultrafast Interfacial Water Dynamics in Intrinsically Disordered Protein Complexes

*Aritra Chowdhury, Sergey A. Kovalenko, Iker Valle Aramburu, Piau Siong Tan, Nikolaus P. Ernsting, and Edward A. Lemke**

anie_201813354_sm_miscellaneous_information.pdf

Supporting Information
Table of Contents

Table of Contents ... 1
Experimental Procedures ... 1
Sample preparation ... 1
Single Molecule Spectroscopy ... 2
Picosecond resolved spectroscopy setup and lifetime measurements ... **Fehler! Textmarke nicht definiert.**
Measuring static microenvironment polarity with acrylodan steady state emission ... 4
Picosecond resolved spectroscopy anisotropy ... 5
Broadband fsTA spectroscopy ... 5
Supplementary Figures and Tables ... 8
References ... 20

Experimental Procedure

Sample preparation

Nup153FG: The codon-optimized (Mr. Gene, Regensburg, Germany) Nup153FG (AA 875–1475 of the human full-length protein, UniProt P49790) was recombinantly expressed in the *Escherichia coli* (*E. coli*) strain BL21(AI) (Thermo Fisher Scientific) following previously described procedures[1]. The labeling mutants with engineered single cysteine mutations (at position 883, 990, 1330, 1355 or 1391; all numbers corresponding to positions in the context of the full length protein) were cloned into a pTXB3-6His-TEV-Int-CBD vector and thus the expressed protein carried an N-terminal 6His tag followed by a TEV protease cleavage site and an intein followed by a chitin binding domain (CBD) at the C-terminal site. Cultures were grown in Terrific Broth (TB) medium at 37˚C, induced with 0.02% arabinose and 1 mM Isopropyl β-D-1-thiogalactopyranoside (IPTG) at OD (600 nm) ~1.0 and harvested by centrifugation after 6-8 hours. The protein was purified under mild denaturing (2 M Urea) conditions on Ni- and chitin-resins following the manufacturer protocols. N- and C-terminal purification tags were cleaved by TEV protease and intein mediated cleavage, induced with 100 mM β-mercaptoethanol respectively. A final round of purification was done on Ni-resins to remove proteins still bearing the 6His tag or the free tag. For smFRET a mutant bearing a stop (TAG) mutation alongside a cysteine mutation (883C/938TAG) was adapted form ref [2]. Briefly, *E. coli* strain BL21(AI) harbouring the pEVOL-PylRS plasmid in presence of 1 mM of PrK (N-Propargyl-L-Lysine) was added at OD ~0.2. The purification of the PrK bearing Nup153FG was identical to the single cysteine labelling mutants.

Importinβ: Importinβ gene (UniProt Q14974) cloned in a pTXB3-Int-12His vector and was expressed as a C-terminal intein-12 His fusion construct in *E. coli* BL21 (AI) (Thermo Fisher Scientific) following previously described procedures[1]. Cultures were grown in TB medium at 37˚C, and induced at OD_{600} ~0.4-0.6 with arabinose and IPTG. After induction, temperature was reduced to 30˚C and growth was continued overnight. The bacteria were harvested and purified under native conditions according to manufacturer's protocols for His-tagged proteins. The C-terminal purification tag was removed by intein cleavage in 100 mM β-mercaptoethanol. A second round of purification was done on Ni-resins to

remove proteins still bearing the 12His tag or the free tag. The protein was finally purified on Superdex 200 (GE Healthcare) column on an Aekta (GE Healthcare) FPLC system.

IBB: IBB (the first 95 residues forming the disordered part of human Importinα-1, UniProt P52292) bearing engineered single (S24C) or double (S24C/S55C) cysteine mutation cloned in pBAD-Int-CBD-12His vector were expressed as C-terminal intein-12His fusion construct in *E. coli* BL21 (AI) (Thermo Fisher Scientific). Cultures were grown in TB medium at 37˚C and induced at OD 0.4-0.6 with arabinose and IPTG. After induction, growth was continued for 5–6 h. Bacteria were harvested and purified under mildly denaturing (Urea 2M) conditions according to standard manufacturers protocols for His-tagged proteins. The C-terminal purification tag was removed by intein cleavage in 100 mM β-mercaptoethanol. A final round of purification was done on Ni-resins to remove proteins still bearing the His12 tag or the free tag.

Labelling: Prior to labelling with thiol reactive dyes, proteins were reduced with 10 mM 1,4-Dithiothreitol (DTT) at room temperature. After reduction the DTT was removed by sequential buffer exchange in an centrifugal filter with 3kDa MW cutoff (Amicon). Labelling with Alexa488,594 or acrylodan (Thermo Fisher Scientific) were performed by incubating >100µM freshly reduced proteins with 5-fold excess dyes in pH 7 buffer (4 M Guanidinium hydrochloride, 1x PBS, 0.1 mM EDTA, 0.2 mM Tris(2-carboxyethyl)phosphine (TCEP)) for two hours (for Alexa488/594 maleimide) or four hours (for acrylodan) at room temperature followed by quenching of the reaction with excess DTT addition (10 mM final concentration). For samples used in smFRET measurements IBB(S4C/S55C) was simultaneously labelled with 5x each of Alexa488 and Alexa594-maleimide and Nup153FG (883C/990TAG) was labelled sequentially with Alexa488 alkyne and Alexa594 maleimide as reported before [2].

Single Molecule Spectroscopy

Single-molecule fluorescence experiments were performed on a home-built multiparameter spectrometer, as described before[3]. The spectrometer was centered around a high-numerical-aperture water-immersion objective (Nikon 60x, 1.27 NA) on a z-translator. Linearly polarized output from two picosecond laser diode (LDH 485 and LDH-DTA-560, Picoquant, Berlin, Germany) was filtered through an excitation filter of 482/18 and 572/15-25, was passed through a linear polarizer and used to excite Donor and Acceptor dyes. The fluorescence emission from the Donor and Acceptor dyes was spatially filtered with a 0.1 mm pinhole, then split into parallel and perpendicular componets each which was subsequently spectrally separated into "green" (Donor) and "orange" (Acceptor) (emission filters 525/50, 612/69) fluorescence components and finally detected with single photon counting detectors. The Donor signal was detected with two PMA Hybrid detector (PicoQuant), while the Acceptor signal was detected using two tau-SPAD single photon avalanche photodiodes (PicoQuant). The laser pulses were alternated in order to probe the presence of the acceptor[4] and laser synchronization was achieved through a multichannel lase driver Sepia II (Picoquant). Photon signals were acquired using a multichannel time-correlated single-photon counting module (Hydraharp400, PicoQuant).

Acquired data were subjected to multiparameter fluorescence detection (MFD) analysis[4a, 5]. Single molecules were identified via a burst search algorithm on the Lee-filtered photon stream and fluorescence intensities (I), lifetimes (τ) and anisotropies (r) were extracted from individual bursts. Data were analyzed with a custom-written program using Igor Pro (Wavemetrics, Lake Oswego, OR). The interphoton lag time threshold for burst selection was set to 160 microseconds and identified bursts were further subjected to a photon based selection criteria of 50 photons. To discard events where acceptor photobleaching occurs during a burst a threshold criterion of 200 µs for the difference in average macroscopic photon arrival times following green and orange excitation was used[4a].

FRET (E_{FRET}) efficiencies in a photon burst were related to the photon count by equation 1.

$$E_{FRET} = \frac{\gamma {I_A}^D}{\gamma {I_D}^D + {I_A}^D}$$ Equation 1

Similarly, stoichiometry (S) of individual bursts were determined by equation 2.

$$S = \frac{\gamma {I_D}^D + {I_A}^D}{\gamma {I_D}^D + {I_A}^D + {I_A}^A}$$ Equation 2

where ${I_X}^Y$ describes the corrected intensity detected from the Donor (D) or Acceptor (A) dye via Donor or Acceptor lasers, i.e., the 488 nm and 561 nm lasers respectively and γ accounts for differential quantum yields of donor and acceptor dyes and differential detection efficiencies of acceptor and donor channels. Raw intensities were corrected for background, leakage of donor signal into the acceptor channel and direct acceptor excitation from donor excitation and γ was estimated from the correlation between S and E_{FRET} as done before [2, 3b].

Measurements were made on ~ 50 -100 pM double labeled samples. PIE allows us to directly monitor the presence of acceptor and subsequently allows us to distinctly identify molecules with only a donor or acceptor dye from those having both Donor and Acceptor dyes in the S vs E_{FRET} 2D histograms (See Figure S1). The molecules having only the Donor dye and lacking an active Acceptor dye show up as a distinct population at S~1 and E_{FRET}~0, while those bearing only acceptor dye show up as a population of variable E_{FRET} and S~0 while molecules bearing both Donor and Acceptor dyes show up as a population with S~0.5. The last one is only used to evaluate E_{FRET} of a given sample and can be filtered from the other populations using their S values. To evaluate only molecules where both dyes are present we only chose bursts within S values of 0.7 to 0.3 and this filtered dataset was used to monitor FRET changes as shown in Figure 1 of maintext.

An advantage of MFD analysis is also that one have access to burstwise lifetimes. Lifetime and FRET efficiencies can be expressed by the equation 3.

$$\tau_{DA} = (1 - E_{FRET})\tau_{DO}$$ Equation 3

where τ_{DA} and τ_{DO} are the donor lifetimes in presence and absence of FRET respectively. From MFD measurements both τ_{DA} and τ_{DO} are readily accessible from the donor only molecules and the FRET molecules respectively. We use maximum likelihood estimator (MLE) to get one lifetime value out of every burst> 60 photons[6]. A plot of Equation 3 on a 2D histogram of donor lifetime vs E_{FRET} efficiency is known as the static line. An offset form the static line is indicative of sub-millisecond dynamics in the sample[7]. This is because on a

smFRET data also gives one access to hydrodynamic diffusion properties of the molecules via fluorescence correlation spectroscopy (FCS) which measure Brownian diffusion induced decay of correlation of intensity fluctuations of fluorescence detected from a small volume (here a confocal excited volume). The correlation function is defined by Equation 4 where $G(\tau)$ is the correlation for lag-time τ where $\delta I(t)$ and $\delta I(t+\tau)$ are the difference in fluorescence intensity from the mean intensity $\langle I \rangle$ at time t and $t+\tau$ respectively.

$$G(\tau) = \frac{\langle \delta I(t)\delta I(t+\tau)\rangle}{\langle I \rangle^2}$$ Equation 4

Since in our setup we use an underfilled objective with a 100 micron large pinhole our elongated confocal volume can be approximated to be two dimensional and thus the FCS data was analysed with a 2D diffusion equation with a triplet state given Equation 5.

$$G(\tau) = \frac{1}{N}\left[1 + \frac{T}{1-T}\exp\left(-\tau/\tau_T\right)\right]\left(1 + \frac{\tau}{\tau_D}\right)^{-1}$$ Equation 5

Here $G(\tau)$ is the correlation for lag-time τ, T is the fraction of molecules in the triplet state and τ_T is the single-triplet relaxation time and τ_D is the diffusion time.

For samples in the bound state, measurements were made in presence of 2 µM Importinβ and for the unbound state measurements were made in the absence of any other protein. All samples were dispersed in 1xPBS buffer (pH-7.4) supplemented with 2 mM magnesium acetate (MgOAc), 10mM DTT and 0.004% Tween-20. To explore the interaction of the two IDPs, IBB and Nup153FG we launched a battery of analysis as mentioned above on the smFRET data to have a holistic picture of the interaction (See Figure S1).

Picosecond resolved spectroscopy setup and lifetime measurements

Ensemble time resolved studies were performed on a home-built (Time Correlated Single Photon Counting) TCSPC spectrometer. The excitation path consisted of a QuixX 405 picosecond pulsed laser (Omicron, Germany) and a polarizer. The emission path perpendicular to the excitation path consisted of a polarizer and a monochromator; signal was finally detected on a PMA Hybrid detector (PicoQuant, Berlin) module coupled to a Hydraharp400 module (PicoQuant, Berlin) for photon counting. Excitation pulses at 400 nm at 30 MHz repetition rate was used to excite the samples. Picosecond resolved measurements were made on 1 µM Nup153FG or IBB free state or in presence of 100 µM and 25 µM Importinβ respectively in 1xPBS buffer (pH-7.4) supplemented with 2mM MgOAc and DTT.

For fluorescence lifetime data, measurements were made under magic angle polarization conditions (where the emission polarizer was set at 54.7° relative to the excitation polarization). The measured decays $I(t)measured$ is as a convolution integral of the instrument response function IRF. The IRF was measured with a 1% ludox (Sigma Aldrich) solution and had a typical full width at half maximum (fwhm) of 140 ps in our setup. The fluorescence decay function $I(t)$ given by equation 6[8].

$$I(t)measured = \int_0^t I(t-t')IRF(t')dt' = I(t)\otimes IRF \qquad \text{Equation 6}$$

The decays were fitted as a convolution integral of the IRF and a sum of exponentials with time constants τ_i and a_i as corresponding amplitudes, which was used to model the *I(t)*, as in equation 7[8].

$$I(t) = \sum_{i=1}^{n} a_i \exp(-\frac{t}{\tau_i}) \qquad \text{Equation 7}$$

Measuring static microenvironment polarity with acrylodan steady state emission

For many solvatochromic probes maximal frequency of emission (ν_{max}) shows a good correlation with the relative permittivity ε_r. Hence to estimate the local polarity expressed by ε_r we used emission maxima data for acrylodan-2-mercaptoethanol conjugate, in different solvents (1,4-dioxane, ethanol, methanol, acetonitrile and water) of different ε_r from literature[9]. The micropolarity in terms of ε_r experienced by acrylodan in the different protein environments measured was estimated from the emission maxima ν_{max} using equation 8, which was obtained from a linear fit of the emission maxima data for acrylodan-2-mercaptoethanol conjugate in different solvents vs corresponding ε_r of those solvents (See example in Figure S5). A very similar approach was used to infer local micropolarity of protein environments using badan, which has the identical chromophore to acrylodan but is short of one methylene linker[10]. We estimate the precision in estimation of relative permittivities to be >± 2.

$$\varepsilon_r = -0.013\nu_{max} + 304 \qquad \text{Equation 8}$$

Picosecond resolved anisotropy decays

For time resolved anisotropy measurements, decays were measured at parallel $(I(t)_{para})$ and perpendicular $(I(t)_{perp})$ polarization conditions with the emission polarization set to 0° and 90° with respect to the excitation polarization respectively. The anisotropy *r(t)* decay was constructed following equation 9[11]

$$r(t) = \frac{I(t)_{para} - GI(t)_{perp}}{I(t)_{para} + 2GI(t)_{perp}} \quad \text{Equation 9}$$

G is a parameter that accounts for the differential detection efficiencies in different polarizations and was obtained using the long-time tail matching method[12] by measuring a small free dye in a low viscosity solvent, in our case Bis-ANS ((4,4'-dianilino-1,1'-binaphthyl-5,5'-disulphonic acid, dipotassium salt) from Thermo Fisher Scientific) dye in methanol. For such a system, which is freely rotating, the tails of the parallel and perpendicular decay becomes equal at long time or in other words the anisotropy decays to zero. The G factor was obtained by varying the G factor until the tail of the anisotropy was centred on zero value. The anisotropy decay is described as a sum of exponentials, given by equation 10[11], with time constants ϕ_i , known as rotational correlation times, having corresponding amplitudes a_i and with the r_0 the initial anisotropy and r_{inf} the residual anisotropy which is used to account for the fact that in the limited time window given by the fluorescence lifetime of the probe anisotropy does not decay to zero.

$$r(t) = (r_0 - r_{inf})[\sum_{i=1}^{n} a_i \exp(-\frac{t}{\phi_i})] + r_{inf} \quad \text{Equation 10}$$

Broadband fsTA spectroscopy

The broadband fsTA spectroscopy setup has been extensively described in our previous work[13]. Transient absorption spectra were recorded following excitation by ~50 fs, 0.6 μJ pump pulses at 400 nm and measured with supercontinum probe pulses generated in a CaF_2 plate. Variable time delays between the pump and probe pulses were obtained by a moving delay stage. The pump beam was chopped at a frequency half of that of the probe pulse to record pump-induced spectral changes. All spectra were recorded with magic angle between the pump and probe polarizations. The transient spectra were time-corrected for the chirp of the supercontinuum. The sample was contained in a modified 1 mm Helma cuvette. The cuvette was moved up-and-down with 2 mm amplitude with a frequency of 1 Hz; it was also oscillated horizontally at 20 Hz. Measurements were carried out in steps of 20 fs (until 6 ps), 400 fs (until 120 ps), and 8 ps (until ~1800 ps). Small (< ±6%) differences of signal intensity between the three data sets were corrected afterwards, by comparing average amplitudes at the regions of overlap. The samples typically had an absorbance of 15-25 mOD at the pump wavelength. For unbound IDPs typically ~100 μM acrylodan IDPs were used and for the bound conditions 100 μM IDPs and ~350 μM Importinβ was used. For most datasets samples were changed 3 times during acquisition to maintain sample integrity. The Time Dependent Stokes Shift (TDSS) was obtained by following the time evolution of the emission band of acrylodan after excitation. The emission band was recognized by a region of negative induced optical density, OD<0, corresponding to Stimulated Emission (SE) in the fluorescence region. Acrylodan was found to have a very prominent SE band that is spectrally well separated from Excited State Absorption (ESA) and ground state bleach (BL) thus circumventing the need of spectral decomposition of the transient spectra. For each time step we determined the mean emission frequency. For this purpose every TA spectrum was first converted into the corresponding distribution of induced oscillator strength, ~OD/ν as a function of probe wavenumber. The emission region in the thus obtained induced oscillator distribution $I(\tilde{\nu}F)$ was then fitted by a (negative-going) lognormal function with some linear background given by equation 11 where $I(\tilde{\nu}F)$ is the induced oscillator strength at frequency $\tilde{\nu}F$, $\tilde{\nu}0$ is the frequency where the induced oscillator strength is maximum, h is the amplitude, Δ is the width, γ the asymmetry and k the linear background.

$$I(\tilde{\nu}F) = he^{[-\ln 2(\frac{[1+2\gamma(\tilde{\nu}F-\tilde{\nu}0)/\Delta}{\gamma})^2]} + k \quad \text{Equation 11}$$

Using this fit function, we determined maximal frequency and the two frequencies at half maxima. Thus one had two guesses for the peak of the emissive oscillator distribution: (i) the peak point, and (ii) the average of the red and blue half points. Of these, the 50%/50% average was formed and taken as the instantaneous mean emission wavenumber (See Figure S8).

Combining fsTA data with TCSPC data to obtain (Time Dependent Stokes Shift) TDSS from femtoseconds to nanoseconds and analysis of solvation dynamics

The dynamic evolution of the TDSS was not completed in the time window of fsTA spectroscopy (See Figure S8). To extend the time window of the TDSS to longer times we complemented the fsTA spectroscopy data with the TCSPC data which offers an extended time window up to 20 ns. The raw TCSPC traces at multiple wavelengths contain the information about the rate of fluorescence decays at different wavelengths. However, this information by itself is not sufficient to reconstruct the spectra at different time points without appropriate normalization of the TCSPC traces. Conventionally this normalization is achieved via reconvolution fitting of the TCSPC traces and normalizing the integral of the decay curve, obtained from the fitting parameters, to the steady state intensity[14]. However broadband fsTA spectroscopy provides the fluorescence oscillator distribution at different times and thus a reference spectra for normalization at different time points. If a TCSPC trace at a given wavelength λ is normalized at a given time point t to the oscillator strength value at λ using the oscillator strength distribution at time t obtained from fsTA spectroscopy, then the normalized TCSPC trace intensity at any time point is proportional to the fluorescence oscillator strength at λ at that time point. Thus, if a set of TCSPC traces are normalized in this way using fsTA obtained fluorescence oscillator distribution, then all such normalized traces would be proportional to the oscillator strengths at the corresponding wavelengths at any given time. Thus, from such a set of normalized TCSPC traces the oscillator strength distribution and thus the fluorescence spectra can be recovered. Preceding such normalization, the time zero of the TCSPC traces needs to be estimated. One approach is to use the position maxima of the Instrument Response Function (IRF), however since the decays were measured at ≥75 nm away from the excitation wavelength while the IRF is measured at the excitation wavelength, timing effects from the optics of the monochromator might render using such a method inaccurate. Alternatively the maxima of each TCSPC trace could be used as the time zero point. This approach was used for λ<steady state emission maximum. For λ>emission maximum due to solvation there can be a rise (a component with a negative pre-exponential factor) and thus the maximum is reached at a time greater than $t=0$. For all the λ>steady state emission maximum for every TCSPC trace at a given wavelength, a monoexponential emitter (Rhodamin6G, R6G) was measured at the same wavelength. The maximum of the R6G trace at a given wavelength was considered to be the time zero of the TCSPC traces of the sample at that wavelength. After estimating the time zero, the TCSPC curves were appropriately normalized (see example in Figure S9). The oscillator strength distribution obtained from the normalized TCSPC traces were fitted to a lognormal line-shape using equation 8 and the frequency maxima of such oscillator distributions as a function of time provided the TDSS at longer times. TCSPC traces at all the measured wavelengths were normalized by the intensity values of the fsTA derived emission spectra at those wavelengths at different time points and the corresponding TDSS obtained were identical albeit having minor vertical offsets between them (>50 cm^{-1}). While we normalized the TCSPC traces at a single time point to fsTA obtained oscillator distribution, TDSS obtained from such TCSPC traces follow the pattern of the TDSS from fsTA data at shorter and longer times than the time point used for normalization (See Figure S9), this validated the self-consistency of the method.

A potential disadvantage of the method is that without reconvolution we do not exploit the full time resolution of TCSPC and can only obtain TDSS from the raw TCSPC traces at time points where the effect of the IRF convolution becomes negligible. This limitation was easily circumvented by the fact that at shorter times we exclusively depend on fsTA spectroscopy, which anyways provide a much higher time resolution; the sole motivation for this approach was based on the pursuit of retaining real experimental noise in the data which is artificially smoothened out by reconvolution based fitting. Finally, TDSS obtained from TCSPC traces normalized at 1000ps or 1500ps to the corresponding fsTA spectra were combined with the fsTA data (see Supplementary Figure S9) after correcting for minor

offsets, where needed (>50cm^{-1}).Such small offsets likely results from minor error in manual setting of the monachromator wavelengths for TCSPC measurements.

The combined TDSS data from fsTA spectroscopy and TCSPC provides dynamics of solvation from femtoseconds to nanoseconds. The data was fitted with a minimalistic empirical power law type function given by equation 12 where $\nu(t)$ is the frequency at a given time t, a and b are constants and n is the powerlaw exponent of time (See Supplementary Figure S10, S11 for examples).

$$\nu(t) = a + bt^{-n} \quad \text{Equation 12}$$

The TDSS data for different conditions had different values of absolute stokes shift and to facilitate comparison a normalized correlation function $S(t)$ was used. $S(t)$ allowed us to compare the rate of TDSS in two systems and is given by equation 13. To construct the $S(t)$ function the stokes shift data was normalized at two time points 0* and t∞, the former is the earliest common experimental time point between two datasets and the latter is the time point where the systems reached a quasi stationary state, that is the stokes shift converged such that at times ≥ t∞ the TDSS reached a constant value. The normalized $S(t)$ has a value of 1 at time 0* and ~0 at times ≥ t∞. In equation 10 $v(0*)$ is the TDSS frequency at time 0* and $v(\infty)$ is the frequency of the quasi-stationary state at times ≥ t∞ where the TDSS converges,typically found to be ≥ 10 ns for all our datasets.

$$S(t) = \frac{v(t) - v(\infty)}{v(0*) - v(\infty)} \quad \text{Equation 13}$$

Supplementary Figures and Tables

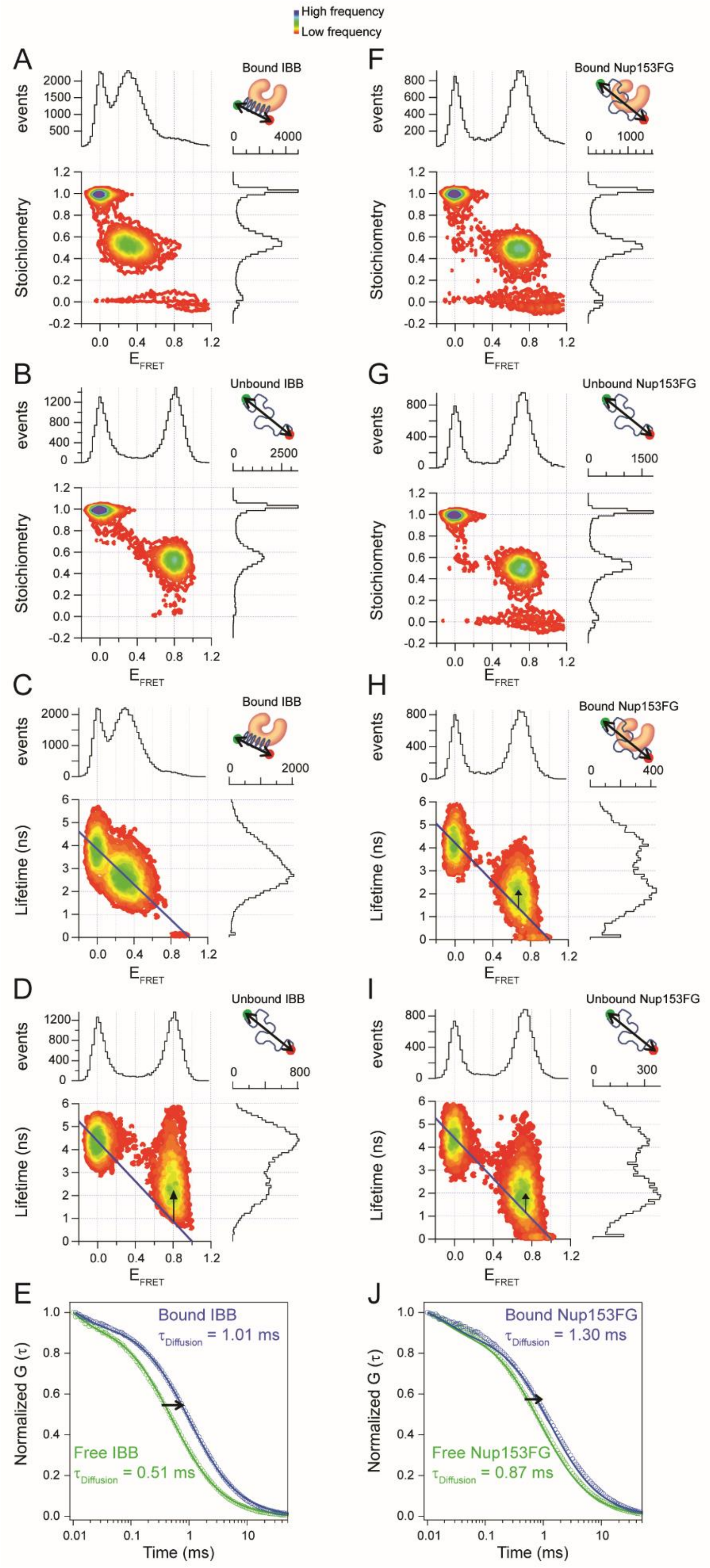

Figure S1. smFRET of IBB-Importinβ and Nup153FG-Imortinβ interaction A-E) smFRET analysis of IBB-Importinβ interaction. A-B) 2D histograms of stoichiometry (S) vs FRET efficiency (E_{FRET}). from measurements done on IBB S24C/S55C double labelled with Alexa488 and Alexa594 maleimide, as donor and acceptor respectively, in presence of 2 µM Importinβ (A) and absence of any protein (B) The plots shows three populations donor only population (S~1, E_{FRET} ~0), an acceptor only population (S~0, variable E_{FRET}) and a doubly labelled population (S~0, variable E_{FRET}). The doubly labelled population carries FRET information and was filtered by selecting events with the criterion 0.7>S>0.3 and analysed (Figure 1 maintext). C-D).The same data as in A (C) and B (D) plotted as a 2D histogram of donor lifetime vs E_{FRET} with an overlay of the static FRET line (equation 3, filtered for S> 0.2 to remove acceptor only signal). The FRET population sits in nearly on the static line in presence of Importinβ (C) but shifts upwards in absence (D) indicating less dynamics in the bound vs the unbound state. E) Normalized FCS from the acceptor fluorescence upon PIE excitation obtained from the smFRET dataset for IBB in presence of Importinβ (blue trace) and its absence (green trace). Solid lines represent fits and the values of the diffusion times are quoted, a slower diffusion time in presence of Importinβ confirms binding. F-J) smFRET analysis of Nup153FG-Importinβ interaction. F-G) 2D histograms of stoichiometry (S) vs FRET efficiency (E_{FRET}). from measurements done on Nup153FG 883C/938TAG double labelled with Alexa488 alkyne and Alexa594 maleimide (see [2] for expeiremntla details on smaple preparation), as donor and acceptor respectively, in presence of 2 µM Importinβ (F) and absence of any protein (G) The plots shows three populations donor only population, an acceptor only population (S~0, and a doubly labelled population. The doubly labelled population carries FRET information and was filtered by selecting events with the criterion 0.7>S>0.3 and analysed (Figure 1 maintext). H-I).The same data as in F (H) and G (I) plotted as a 2D histogram of donor lifetime vs E_{FRET} with an overlay of the static FRET line (equation 3, filtered for S> 0.2 to remove acceptor only signal). The FRET population shifts upwards both in presence (H) and absence (I) of Importinβ dynamics in both the bound and the unbound state. J) Normalized FCS from the acceptor fluorescence upon PIE excitation obtained from the smFRET dataset for Nup153FG in presence of Importinβ (blue trace) and its absence (green trace). Solid lines represent fits and the values of the diffusion times are quoted, a slower diffusion time in presence of Importinβ confirms binding

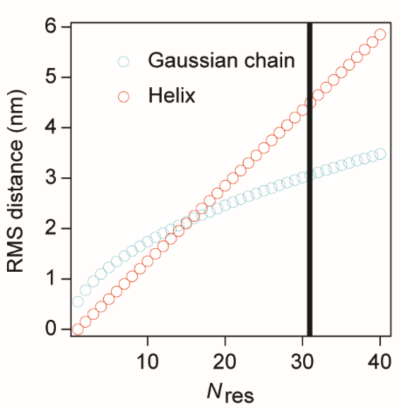

Figure S2. Root Mean Square (RMS) distance of a model Gaussian chain and a Helix to validate conformation change in IBB The RMS end to end distance is plotted as a function of the number of residues (N_{res}) for a Helix (red circles) and a statistical coil (cyan circles). The black line represents the N_{res} value for IBB smFRET construct and shows that for that sequence length a Gaussian chain is shorter than the Helix. For a statistical coil representation of free IBB, we chose a Gaussian chain model with a Flory exponent v=0.5, based on previous our studies[3b], such that RMS(nm)=$0.63N_{res}^{0.5}$ while the RMS distance of a Helix is a linear function of N_{res} such that RMS(nm)=$0.15(N_{res} - 1)$[15]. At a residue separation of 31 aa, which was the separation between the FRET labels in IBB, the RMS distance of a helix happened to be ~1.5 nm longer than that of a Gaussian chain.

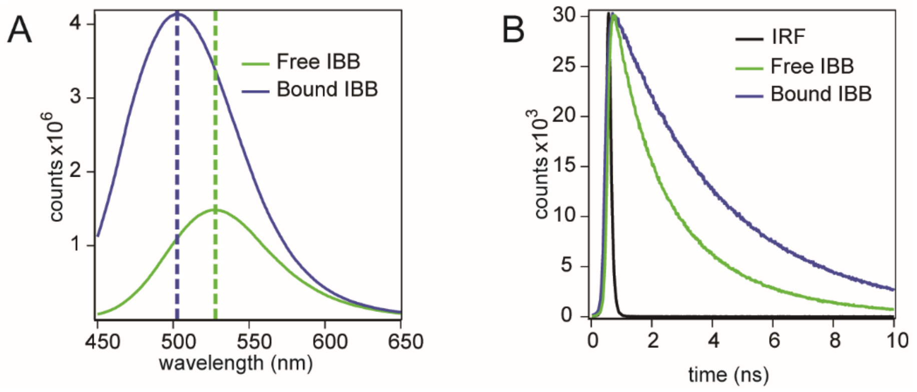

Figure S3. Steady state and time resolved fluorescence for IBB-Importinβ recognition A) Fluorescence spectra of IBB S24C labelled with acrylodan in presence (blue) and absence of Importinβ (green) showing the characteristic blue shift upon binding to the NTR. B) Fluorescence lifetime traces of IBB S24C labelled with acrylodan in presence and absence of Importinβ at 535 nm showing an increase in the lifetime in the presence of Importinβ indicating a hydrophobic environment/binding interface mirroring the steady state results.

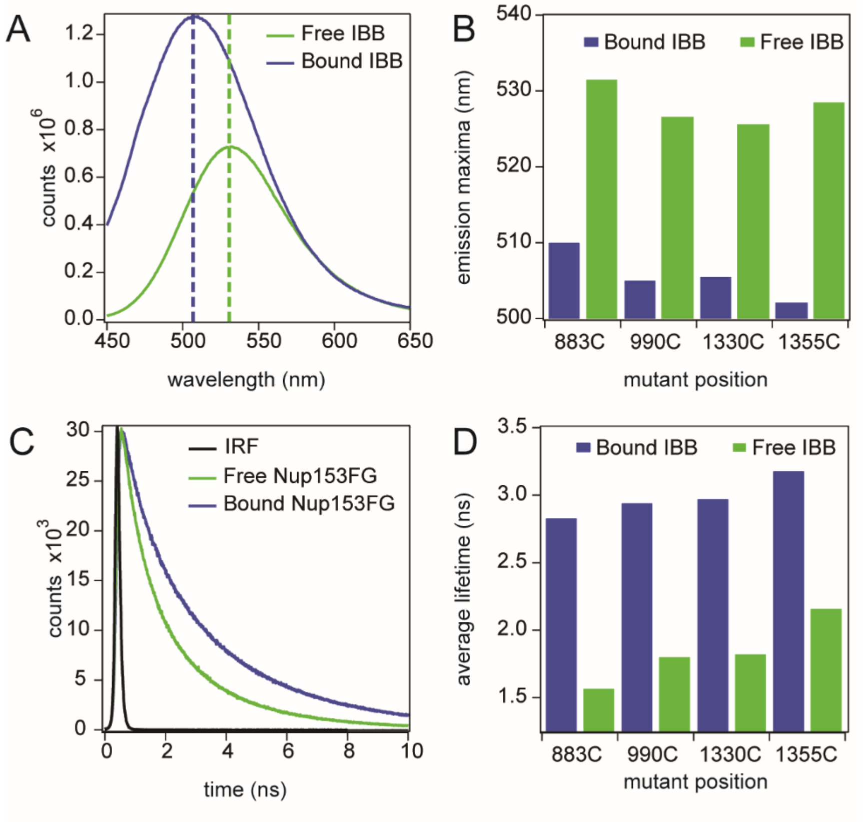

Figure S4. Steady state and time resolved fluorescence for Nup153FG-Importinβ recognition A) Representative fluorescence spectra of Nup153FG 883C labelled with acrylodan in presence (blue) and absence of Importinβ (green) showing the characteristic spectral blue shift upon binding to the NTR. B) The emission maxima of all 4 labelling sites (883,990,1330 and 1355C) in Nup153FG labelled with acrylodan in presence (blue) and absence of Importinβ (green) showed blue shifted emission in presence of Importinβ suggesting a hydrophobic interface. C) Representative fluorescence lifetime traces of Nup153FG 883C labelled with acrylodan in presence (blue) and absence (green) of Importinβ at 530 nm. D) The average fluorescence lifetime of all the 4 labelling sites (883,990, 1330 and 1355 C) in Nup153FG labelled with acrylodan in presence (blue) and absence of Importinβ (green) showed an increase in the average lifetime in the presence of Importinβ. We estimate the upper bounds of the error to be ± 2.5 nm (our emission slit widths) for the emission maxima and ± 300 ps (2 times our IRF fwhm) for average lifetimes respectively.

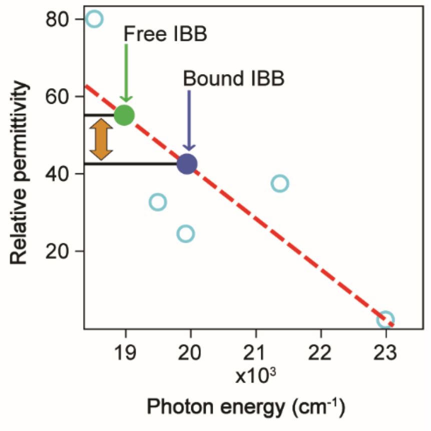

Figure S5. Relative permittivity for IBB in presence and absence of Importinβ. The peak frequencies (cyan open circles) of acrylodan-2-mercaptoethanol conjugate in different solvents[9] is plotted against the relative permittivity of those samples. The red dashed line represents a linear fit through the data. The relative permittivity experienced by acrylodan in unbound IBB (green solid circle) and in presence of Importinβ (blue solid circle) is obtained from the emission peak frequencies using the equation 5. The data indicated decreased relative permittivity of IBB bound to Importinβ compared to free IBB.

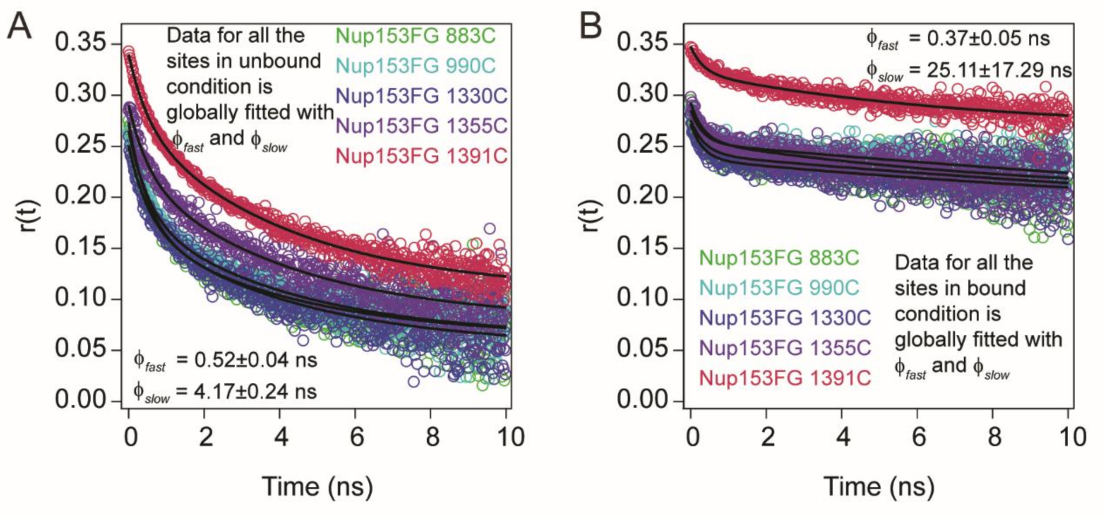

Figure S6. Time resolved anisotropy of Nup153FG-Importinβ interaction A-B) Time resolved anisotropy decay of acrylodan labelled mutants of Nup153FG mutants in absence of Importinβ (A) and in presence of Importinβ (B). The open circles show the raw data for 883C (green), 990C (cyan), 1330C (blue), 1355C (purple) and 1391C (red). The black solid lines represent global fits to equation 7 using two rotational correlation times (ϕ_{fast}, a subnanosecond time component and ϕ_{slow}, a nanosecond time component) which were fitted as two global parameters while the other parameters were allowed to float. All the datasets in absence of Importinβ were globally fitted together in (A) and the same was done for those in presence of Importinβ (B). The values of ϕ_{fast} and ϕ_{slow} are shown for (A) and (B). While the residual anisotropy r_{inf} varies across the samples, it was always higher in the bound vs unbound state (See Table S1).

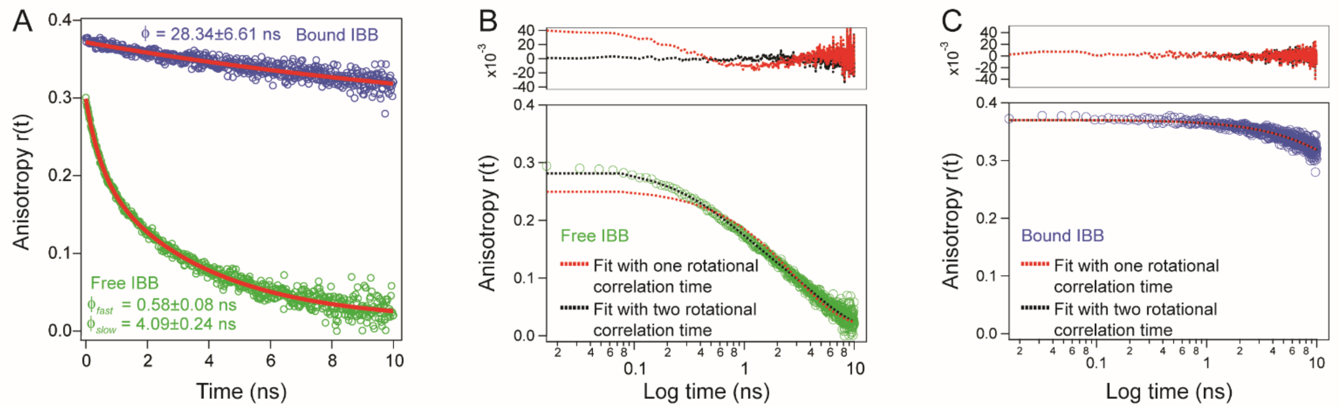

Figure S7. Time resolved anisotropy of Nup153FG-Importinβ interaction A) shows time resolved anisotropy decay of acrylodan labelled IBB S24C in absence of Importinβ (green open circles) and in presence of Importinβ (blue open circles). The data was fitted to equation 7 (red solid lines) using two rotational correlation times (ϕ_{fast} and ϕ_{slow}) for IBB in absence of Importinβ and only one rotational correlation time (ϕ) in presence of Importinβ. Values of the two rotational correlation times ϕ_{fast} and ϕ_{slow} for IBB in absence of Importinβ and that for only one rotational correlation time ϕ for IBB in pressence of Importinβ are shown. B) shows the time resolved anisotropy decay of acrylodan labelled IBB S24C in absence of Importinβ, same as green open circles in (A), fitted to equation 7 with one rotational correlation time (red dashed line) and two rotational correlation times (black dashed lines). The fit with one rotational correlation time deviates from the data and the residual showed distinct patterns while that with two rotational correlation time described the data much better and gave almost featureless residuals, suggesting this to be the most appropriate fitting model. C) shows the time resolved anisotropy decay of acrylodan labelled IBB S24C, same as blue open circles in (A), in presence of Importinβ fitted to equation 7 with one rotational correlation time (red dashed line) and two rotational correlation times (black dashed lines). The fits with one and two rotational correlation times and the corresponding residuals are practically indistinguishable. The fit with two rotational correlation times yields two very closely spaced rotational correlation time values which are individually very similar to that from a fit with one rotational correlation time, suggesting one rotational correlation time to be sufficient and thus the most appropriate fitting model to describe the data.

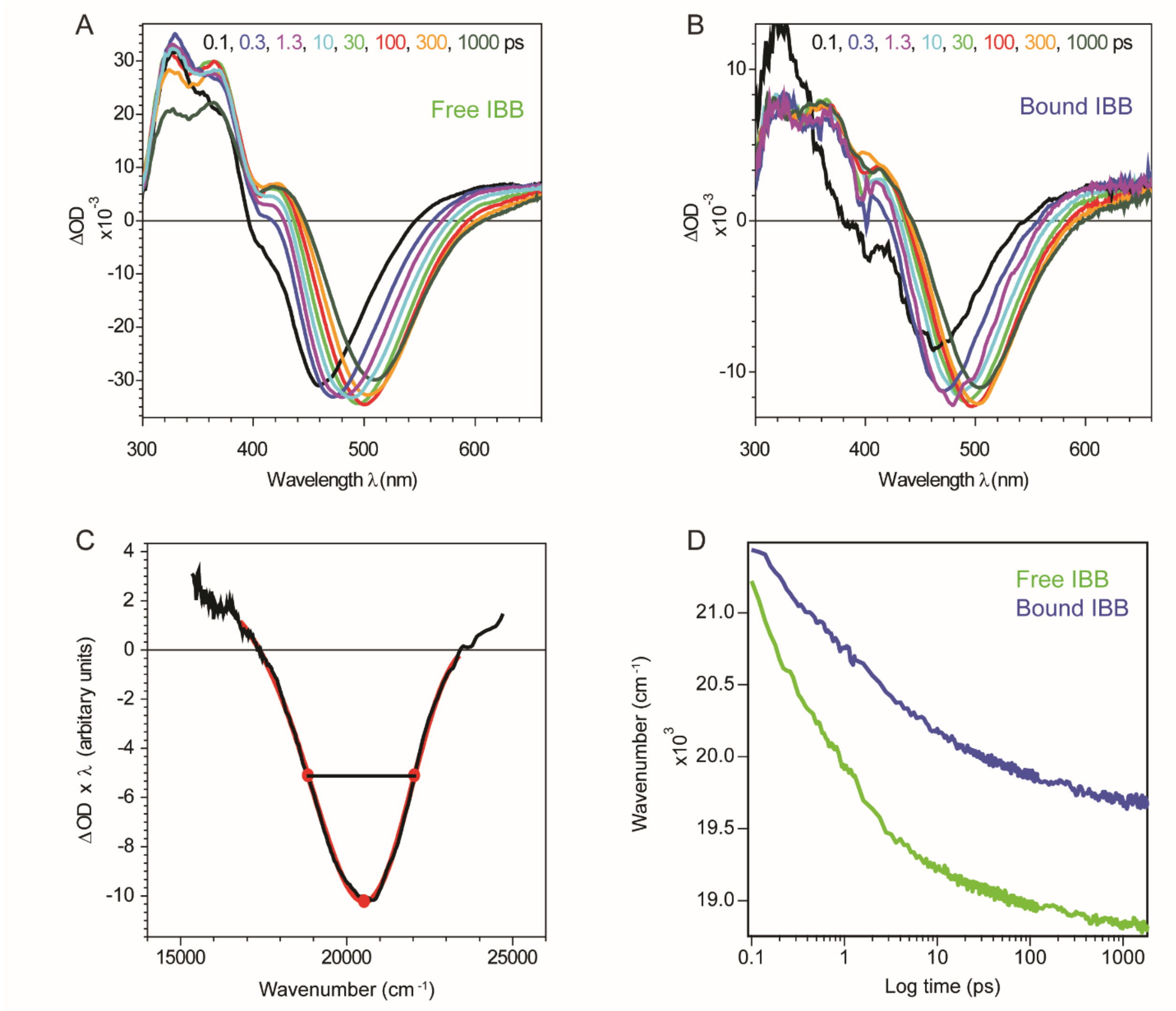

Figure S8. Representative fsTA spectroscopy data A-B) fsTA spectra of acrylodan labelled IBB S24C in absence (A) and in presence of (B) Importinβ recorded for the delay times indicated. The colour of the traces correspond to the colour of the delay times indicated in the figure. (C) Representative example of how maxima of the Stimulated Emission (SE) band was obtained from fsTA data. The SE band (black line) in the oscillator distribution from fsTA spectra of IBB in the absence of Importinβ at 0.3 ps (blue trace in A) was fitted to a lognormal lineshape (red line) as per equation 8. One red dot indicates the maximal frequency and the two red dots joined by a black line represent the frequencies having half maximal value. The emission frequency is taken to be an average of the maximal frequency and an average of the two half maximal frequency. D) TDSS data for IBB in absence (green) and presence (blue) of Importinβ obtained from TDSS data.

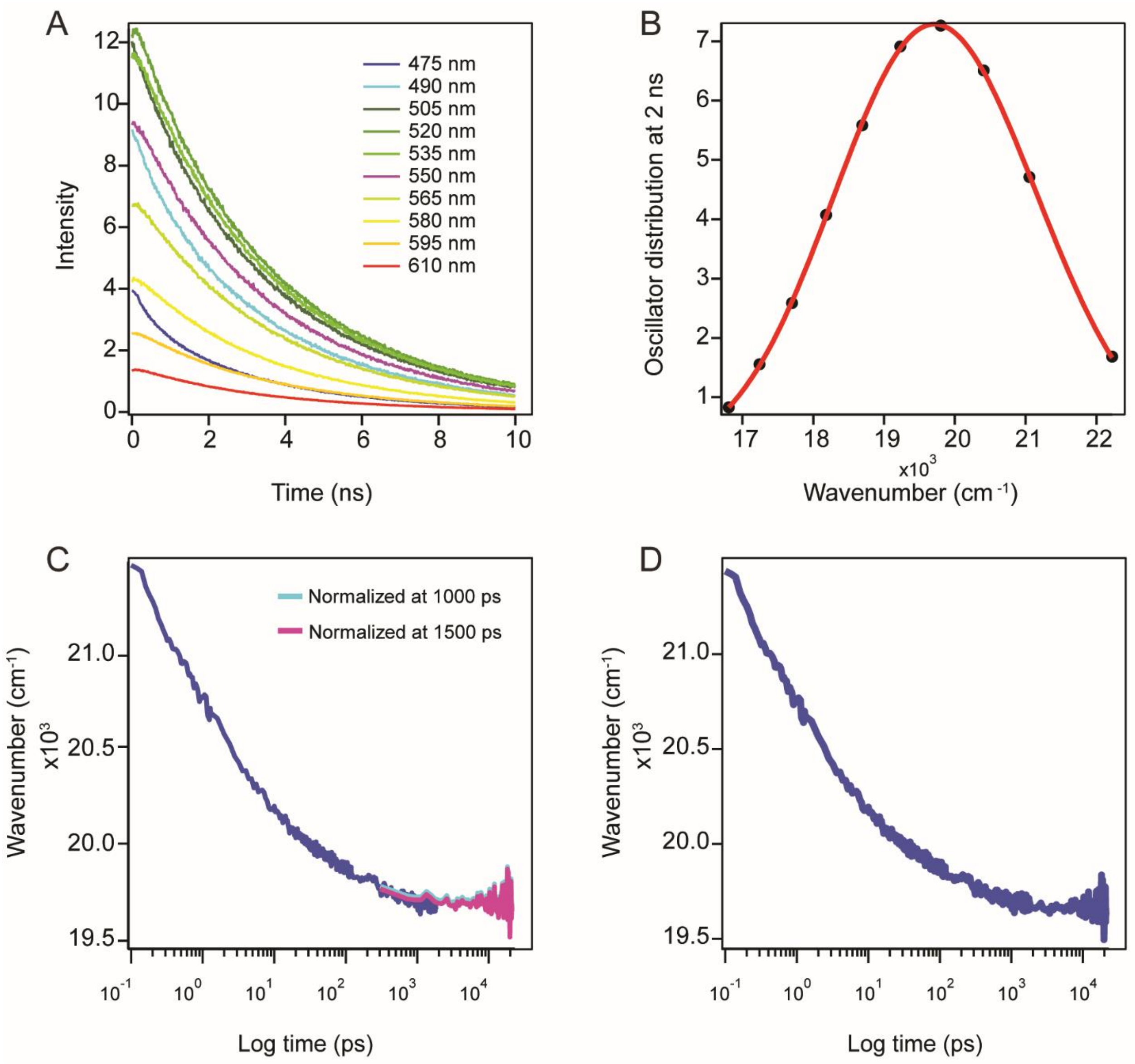

Figure S9. TDSS from femtoseconds to nanoseconds by combining TDSS with TCSPC data A) Representative TCSPC traces at different emission wavelengths (see legend) of IBB S24C in presence of Importinβ normalized to the oscillator strength distribution at 1000 ps obtained from fsTA spectroscopy. B) Oscillator strength distribution obtained from the normalized TCSPC traces (A) at 2 ns fitted to lognormal lineshape (equation 8). C) TDSS data for IBB in presence of Importinβ obtained from TCSPC traces normalized to fluorescence oscillator distributions from fsTA spectroscopy at 1000 ps (cyan), and 1500 ps (magenta) overlaid on the TDSS data obtained from fsTA spectroscopy (blue line). D) Continuous TDSS data for IBB in the presence of Importinβ from 100 fs to 20 ns from combined fsTA and TCSPC data (C) obtained after correcting for a small systematic offset between TDSS data from fsTA spectroscopy and TCSPC.

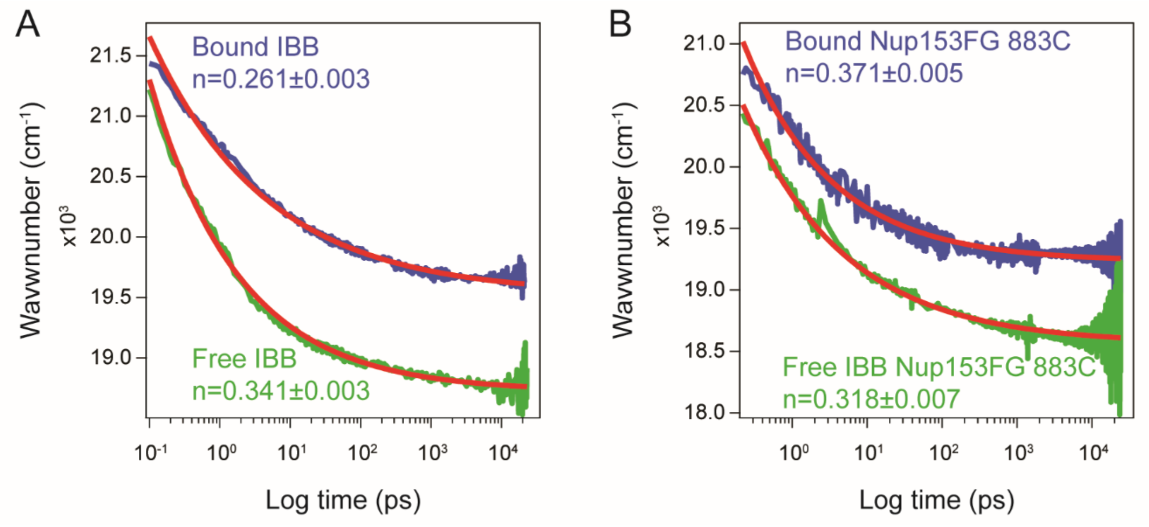

Figure S10. Powerlaw type relaxation of solvation in IDP and IDP complexes A) TDSS data for acrylodan labelled IBB from femtoseconds to nanoseconds in absence (green) and presence of Importinβ (blue) fitted to an empirical powerlaw as given in equation 9 (red lines). The results for the powerlaw exponent *n* are given in the figure for the different conditions. B) TDSS data for acrylodan labelled Nup153FG 883C from femtoseconds to nanoseconds in absence (green) and presence of Importinβ (blue) fitted to an empirical powerlaw as given in equation 9 (red lines).

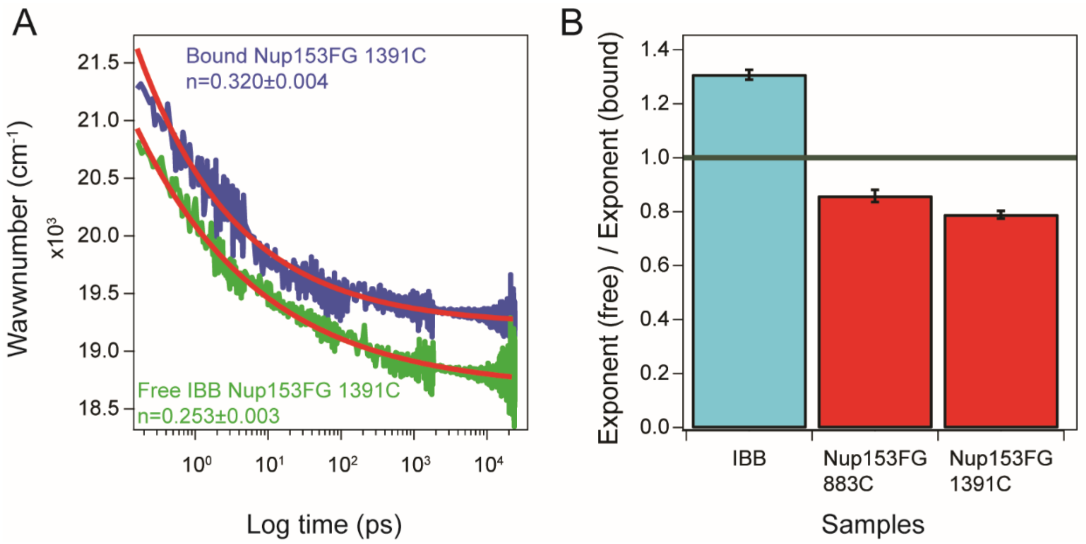

Figure S11. Relaxation of solvation in Nup153FG 1391C and Nup153FG 1391C complex A)TDSS data for acrylodan labelled Nup153FG from femtoseconds to nanoseconds in absence (green) and presence of Importinβ (blue) fitted to an empirical powerlaw as given in equation 9 (red lines) is shown. The results of the powerlaw exponent *n* are given in the figure for the different conditions. B) Ratio of the exponents of powerlaw fits for unbound and the bound states in for Nup153FG 1391C (data in A) is compared with that of Nup153FG 883C and for IBB. Nup153FG mutants are shown as red bars and IBB as cyan bar.

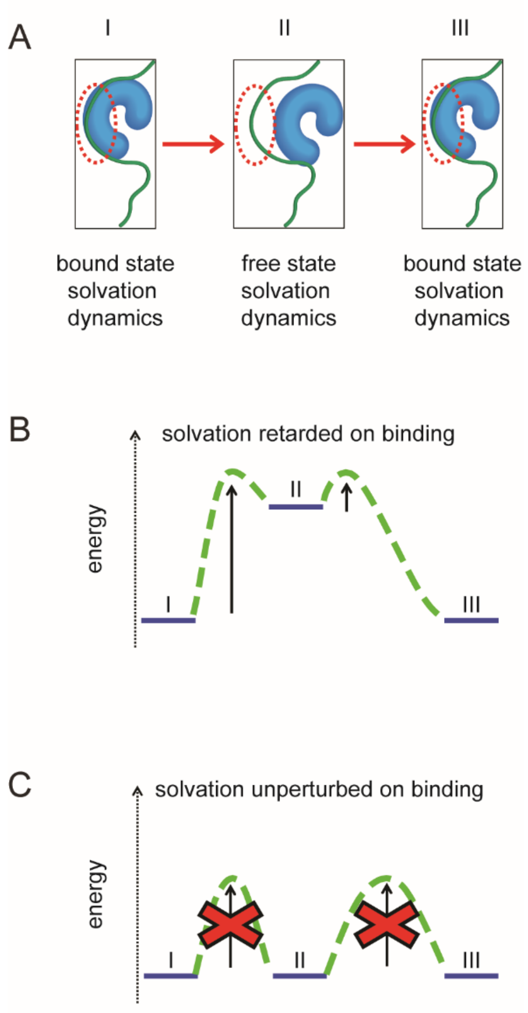

Figure S12. A schematic toy model showing the potential of solvation dynamics modulation in regulation of IDP complex plasticity A) A schematic showing an IDP bound to its folded partner undergoing conformational fluctuations starting from a bound state (I) and a state where there is transient local unbinding of a part of the IDP (II) and again going back to a bound state identical to (I) or a new one (III) but equally stabilized as (I), as can happen in a multivalent system. The circled area represents a selected portion of the IDP that transiently disassociates and re-associates with the partner (and that can be selectively studied using solvation measurements). B) Case A: Schematic showing energy barriers arising from changes in solvation dynamics of the IDP in conformational fluctuation of an IDP when solvation in the bound state is slower than that in the unbound state. The two states described in (A) are represented in terms of energy and are at different free energy levels due to the solvation retardation which results in formation of energy barriers that kinetically impede conformational fluctuations. C) Case B: A schematic similar to that of (C); but in this case solvation dynamics remain unperturbed upon binding. This results in minimal energy differences between the states during the conformational fluctuation making the process barrier-less and thus very fast. Note, our schematic applies to energy barriers arising from reorganization of water dynamics; other barriers may or may not be associated in a context specific way, depending on the partners involved.

Samples	ϕ_{fast} by global fitting all mutants in bound or unbound state	ϕ_{slow} by global fitting all mutants in bound or unbound state	r_0 (initial anisotropy)	r_{inf} (residual anistropy)	α_{fast} (amplitude of the fast component)	α_{slow} (amplitude of the fast component)
Nup153FG 883C unbound	0.52±0.04	4.17±0.24	0.273	0.0512	0.49	0.51
Nup153FG 990C unbound	0.52±0.04	4.17±0.24	0.265	0.062	0.4	0.6
Nup153FG 1330C unbound	0.52±0.04	4.17±0.24	0.251	0.054	0.38	0.62
Nup153FG 1355C unbound	0.52±0.04	4.17±0.24	0.290	0.079	0.31	0.69
Nup153FG 1391C unbound	0.52±0.04	4.17±0.24	0.340	0.108	0.28	0.72
Nup153FG 883C bound	0.37± 0.05	25.11±17.29	0.276	0.156	0.27	0.73
Nup153FG 990C bound	0.37± 0.05	25.11±17.29	0.283	0.165	0.25	0.75
Nup153FG 1330C bound	0.37± 0.05	25.11±17.29	0.271	0.154	0.29	0.71
Nup153FG 1355C bound	0.37± 0.05	25.11±17.29	0.293	0.146	0.28	0.72
Nup153FG 1391C bound	0.37± 0.05	25.11±17.29	0.349	0.198	0.21	0.79

Table S1: Parameters from global fitting of anisotropy decays at 5 sites in Nup153FG in presence and absence of Importinβ. Two rotational correlation times ϕ_{fast} and ϕ_{slow} were fitted globally for all the mutants in the bound and unbound states while all other parameters were allowed to float. The errors are only quoted for the globally fitted parameters as has been discussed in the text.

References

[1] a) S. Milles, D. Mercadante, I. V. Aramburu, M. R. Jensen, N. Banterle, C. Koehler, S. Tyagi, J. Clarke, S. L. Shammas, M. Blackledge, F. Gräter, E. A. Lemke, *Cell* **2015**, *163*, 734-745; b) S. Milles, E. A. Lemke, *Angewandte Chemie* **2014**, *53*, 7364-7367.

[2] S. Milles, S. Tyagi, N. Banterle, C. Koehler, V. VanDelinder, T. Plass, A. P. Neal, E. A. Lemke, *Journal of the American Chemical Society* **2012**, *134*, 5187-5195.

[3] a) P. S. Tan, I. V. Aramburu, D. Mercadante, S. Tyagi, A. Chowdhury, D. Spitz, S. L. Shammas, F. Gräter, E. A. Lemke, *Cell Reports* **2018**, *22*, 3660-3671; b) G. Fuertes, N. Banterle, K. M. Ruff, A. Chowdhury, D. Mercadante, C. Koehler, M. Kachala, G. Estrada Girona, S. Milles, A. Mishra, P. R. Onck, F. Gräter, S. Esteban-Martín, R. V. Pappu, D. I. Svergun, E. A. Lemke, *Proceedings of the National Academy of Sciences* **2017**, *114*, E6342.

[4] a) V. Kudryavtsev, M. Sikor, S. Kalinin, D. Mokranjac, C. A. M. Seidel, D. C. Lamb, *Chemphyschem : a European journal of chemical physics and physical chemistry* **2012**, *13*, 1060-1078; b) B. K. Müller, E. Zaychikov, C. Bräuchle, D. C. Lamb, *Biophysical journal* **2005**, *89*, 3508-3522.

[5] E. Sisamakis, A. Valeri, S. Kalinin, P. J. Rothwell, C. A. M. Seidel, in *Methods in Enzymology, Vol. Volume 475* (Ed.: G. W. Nils), Academic Press, **2010**, pp. 455-514.

[6] J. Schaffer, A. Volkmer, C. Eggeling, V. Subramaniam, G. Striker, C. A. M. Seidel, *The Journal of Physical Chemistry A* **1999**, *103*, 331-336.

[7] S. Kalinin, A. Valeri, M. Antonik, S. Felekyan, C. A. M. Seidel, *The Journal of Physical Chemistry B* **2010**, *114*, 7983-7995.

[8] J. R. Lakowicz, in *Principles of Fluorescence Spectroscopy* (Ed.: J. R. Lakowicz), Springer US, Boston, MA, **2006**, pp. 97-155.

[9] F. G. Prendergast, M. Meyer, G. L. Carlson, S. Iida, J. D. Potter, *J Biol Chem* **1983**, *258*, 7541-7544.

[10] E. Fischermeier, P. Pospíšil, A. Sayed, M. Hof, M. Solioz, K. Fahmy, *Angewandte Chemie International Edition* **2016**, *56*, 1269-1272.

[11] J. R. Lakowicz, in *Principles of Fluorescence Spectroscopy* (Ed.: J. R. Lakowicz), Springer US, Boston, MA, **2006**, pp. 383-412.

[12] M. Tramier, K. Kemnitz, C. Durieux, J. Coppey, P. Denjean, R. B. Pansu, M. Coppey-Moisan, *Biophysical journal* **2000**, *78*, 2614-2627.

[13] A. L. Dobryakov, S. A. Kovalenko, A. Weigel, J. L. Pérez-Lustres, J. Lange, A. Müller, N. P. Ernsting, *Review of Scientific Instruments* **2010**, *81*, 113106.

[14] J. R. Lakowicz, in *Principles of Fluorescence Spectroscopy* (Ed.: J. R. Lakowicz), Springer US, Boston, MA, **2006**, pp. 237-276.

[15] A. M. Melo, J. Coraor, G. Alpha-Cobb, S. Elbaum-Garfinkle, A. Nath, E. Rhoades, *Proceedings of the National Academy of Sciences* **2016**, *113*, 14336-14341.
